# Expert consensus on the diagnosis, treatment, and prevention of respiratory syncytial virus infections in children

**DOI:** 10.1007/s12519-023-00777-9

**Published:** 2023-12-08

**Authors:** Xian-Li Zhang, Xi Zhang, Wang Hua, Zheng-De Xie, Han-Min Liu, Hai-Lin Zhang, Bi-Quan Chen, Yuan Chen, Xin Sun, Yi Xu, Sai-Nan Shu, Shun-Ying Zhao, Yun-Xiao Shang, Ling Cao, Yan-Hui Jia, Luo-Na Lin, Jiong Li, Chuang-Li Hao, Xiao-Yan Dong, Dao-Jiong Lin, Hong-Mei Xu, De-Yu Zhao, Mei Zeng, Zhi-Min Chen, Li-Su Huang

**Affiliations:** 1 Department of Infectious Disease, Children’s Hospital, Zhejiang University School of Medicine, 3333 Binsheng Road, Binjiang District, Hangzhou, 310052 China; 2 Clinical Research Unit, Xin Hua Hospital Affiliated to Shanghai Jiao Tong University School of Medicine, Shanghai, China; 3 Beijing Pediatric Research Institute, Beijing Children’s Hospital, Capital Medical University, Beijing, China; 4 Department of Pediatric Pulmonology, West China Second University Hospital, Sichuan University, Chengdu, China; 5 Department of Pediatric Pulmonology, the Second Affiliated Hospital & Yuying Children’s Hospital, Wenzhou Medical University, Wenzhou, China; 6 Department of Infectious Disease, Anhui Provincial Children’s Hospital, Hefei, China; 7 Department of Pediatrics, the Second Hospital of Hebei Medical University, Shijiazhuang, China; 8 Department of Pediatrics, Xijing Hospital, the Fourth Military Medical University, Xi’an, China; 9 Department of Infectious Disease, Guangzhou Women and Children’s Medicine Center, Guangzhou Medicine University, Guangzhou, China; 10 Department of Pediatrics, Tongji Hospital, Tongji Medical College, Huazhong University of Science and Technology, Wuhan, China; 11 Department of Respiratory Disease, Beijing Children’s Hospital, Capital Medical University, Beijing, China; 12 Department of Pediatric Respiratory, Shengjing Hospital of China Medical University, Shenyang, China; 13 Respiratory Department, Children’s Hospital Affiliated to Capital Institute of Pediatrics, Beijing, China; 14 Department of Clinical Epidemiology, Aarhus University, Aarhus, Denmark; 15 Department of Respirology, Children’s Hospital of Soochow University, Suzhou, China; 16 Department of Respiratory, Children’s Hospital of Shanghai, Children’s Hospital Affiliated to Shanghai Jiao Tong University School of Medicine, Shanghai, China; 17 Department of Infectious Disease, Hainan Women and Children’s Medical Center, Haikou, China; 18 Department of Infectious Disease, Children’s Hospital of Chongqing Medical University, Chongqing, China; 19 Department of Respiratory, Children’s Hospital of Nanjing Medical University, Nanjing, China; 20 Department of Infectious Diseases, Children’s Hospital of Fudan University, 399 Wanyuan Road, Minhang District, Shanghai, 201102 China; 21 Department of Respiratory Diseases, Children’s Hospital, Zhejiang University School of Medicine, 3333 Binsheng Road, Binjiang District, Hangzhou, 310052 China

**Keywords:** Consensus prevention, Respiratory syncytial virus, Treatment

## Abstract

**Background:**

Respiratory syncytial virus (RSV) is the leading global cause of respiratory infections and is responsible for about 3 million hospitalizations and more than 100,000 deaths annually in children younger than 5 years, representing a major global healthcare burden. There is a great unmet need for new agents and universal strategies to prevent RSV infections in early life. A multidisciplinary consensus development group comprising experts in epidemiology, infectious diseases, respiratory medicine, and methodology aims to develop the current consensus to address clinical issues of RSV infections in children.

**Data sources:**

The evidence searches and reviews were conducted using electronic databases, including PubMed, Embase, Web of Science, and the Cochrane Library, using variations in terms for “respiratory syncytial virus”, “RSV”, “lower respiratory tract infection”, “bronchiolitis”, “acute”, “viral pneumonia”, “neonatal”, “infant” “children”, and “pediatric”.

**Results:**

Evidence-based recommendations regarding diagnosis, treatment, and prevention were proposed with a high degree of consensus. Although supportive care remains the cornerstone for the management of RSV infections, new monoclonal antibodies, vaccines, drug therapies, and viral surveillance techniques are being rolled out.

**Conclusions:**

This consensus, based on international and national scientific evidence, reinforces the current recommendations and integrates the recent advances for optimal care and prevention of RSV infections. Further improvements in the management of RSV infections will require generating the highest quality of evidence through rigorously designed studies that possess little bias and sufficient capacity to identify clinically meaningful end points.

## Introduction

In the past decade, the substantial burden of respiratory syncytial virus (RSV) has attracted global attention. RSV is associated with about 33 million cases of lower respiratory tract infections (LRTIs), three million hospitalizations, and over 100,000 deaths in children younger than 5 years each year globally, and no decline in morbidity, hospitalization, or mortality has been observed over time [1, 2]. Infants in the first 6 months of life are particularly vulnerable, with a mortality rate of 3.6% attributable to RSV [1]. RSV is the most common reason for infant hospitalization in high-income countries, and it causes a disproportionate number of deaths in low- and middle-income countries [1]. There is, however, a scarcity of consensus or guidelines for the management and prevention of RSV infections in children globally. Previous guidelines focused on bronchiolitis have helped clinicians manage RSV infections to some extent. Nevertheless, there are emerging evidences of distinct mechanistic pathways employed by various viruses causing bronchiolitis, and these differences can be responsible for some of the heterogeneities observed in therapeutic interventions. Therapeutic management tailored to a virological diagnosis is an area for further study. Furthermore, despite two decades of evidence suggesting that less treatment is preferable and advising supportive rather than interventional therapy, the elimination of interventional care has not been achieved globally and remains a major challenge. With advancements in virology, significant progress has been made in the epidemiology, diagnosis, treatment, and prevention of RSV infections. To date, dramatic alternations in the epidemiologic profile of RSV have been reported as a result of the severe acute respiratory syndrome coronavirus 2 (SARS-CoV-2) pandemic [3–7]. The introduction of nonpharmaceutical interventions (NPIs) led first to a sharp decline in global mortality from RSV infections and second to a resurgence of RSV when NPIs had been lifted, which ultimately disrupted the routine and historical seasonality and subsequently caused peaks in atypical periods of the year, thus leading to a considerable impact on global healthcare systems. In addition to palivizumab and nirsevimab, several candidate monoclonal antibodies targeting RSV are currently in the pipeline. Moreover, breakthroughs have been made in RSV vaccines. Therefore, experts in epidemiology, infectious diseases, respiratory medicine, and methodology jointly developed the present consensus, synthesizing the available evidence to better guide clinical practice. The consensus applies to children younger than 5 years, focusing on the most recent research advancements in the epidemiology, clinical manifestations, diagnosis, treatment, and prevention of RSV infections.

## Methods

In January 2023, a steering committee meticulously assembled a consensus development group, including 25 specialists with clinical and/or research expertise in epidemiology, infectious diseases, respiratory medicine, and methodology. The composition of the 25 members was carefully designed to ensure representation from various geographic regions of China, including Beijing, Shanghai, Guangdong, Chongqing, Hebei, Liaoning, Jiangsu, Zhejiang, Anhui, Hubei, Hainan, Sichuan, and Shanxi. All members were free of financial or intellectual conflicts of interest and were granted unrestricted involvement.

The evidence searches and reviews were conducted in January 2023 using electronic databases, including PubMed, Embase, Web of Science, and the Cochrane Library. On these websites, we searched for articles without date restrictions, using variations in terms for “respiratory syncytial virus”, “RSV”, “lower respiratory tract infection”, “bronchiolitis”, “acute”, “viral pneumonia”, “neonatal”, “infant” “children”, and “pediatric”. Furthermore, a comprehensive search was conducted to uncover additional pertinent literature by examining the references of the selected publications. References were regularly updated during the drafting of the consensus.

Reviewers collaborated in pairs, independently performed reference screening and data extraction, and resolved any disagreements through discussion or consultation with a third reviewer. A draft version of the document underwent a thorough evaluation process by consensus development group members. The resulting comments were reviewed by consensus development group members and subsequently integrated into the final draft as appropriate.

A Delphi method was adopted to develop a consensus of pertinent statements. The consensus development group members were requested to vote on each statement of the Delphi questionnaire according to a five-point Likert scale (strongly agree/agree/neither agree nor disagree/disagree/strongly disagree) and provide open text comments, as appropriate. Consensus agreement was defined as an agreement by a minimum of 75% of the participants (i.e., 75% agree or strongly agree). The Delphi questionnaire was completed by all 25 experts via an online survey in July 2023, and final drafted recommendations were formulated. Recommendations that achieved consensus were compiled and then presented.

## Results

### Disease burden

*Recommendations*: RSV substantially contributes to the morbidity and mortality burden globally in children younger than 5 years, particularly during the first 6 months of life. Geographical area, climate, economic status, and nonpharmaceutical interventions affect the seasonality and dynamics of RSV. Epidemiological surveillance of RSV infections in the pediatric population should be conducted proactively.

Human RSV is the predominant pathogen identified in children younger than 5 years with LRTIs [1, 8–13]. RSV strains are classified into subtypes A or B based on the genetic variability of the second hypervariable 2 region of the *G* gene, and these subtypes cocirculate with alternating dominance annually [14]. There were about 33.0 million RSV-LRTI episodes, 3.6 million RSV-LRTI hospital admissions, and 101,400 RSV-attributable overall deaths globally in children younger than 5 years in 2019. The estimated global incidence rate of RSV-LRTIs is 48.8 per 1000 children annually, with variations between developed and developing countries (24.3/1000 vs. 51.6/1000) [1]. Infants aged 0–6 months are at the greatest risk for RSV-LRTIs, with one in five RSV-LRTI episodes, 39% of RSV-LRTI hospitalizations, and 45% of RSV-attributable deaths occurring within this specific age group of infants [1]. The mortality rate also peaks during the first 6 months of life, with RSV being responsible for 3.6% of deaths in children aged 0–6 months [1]. Low- and middle-income countries account for > 95% of RSV-LRTI episodes and > 97% of RSV-attributable deaths and RSV-LRTI in-hospital deaths, with disadvantaged economic status as a substantial risk factor [1]. It is noteworthy that mere 26% of RSV-attributable deaths in children younger than 5 years occurred within hospital settings, which is even more pronounced in low-income countries, as only 19% of the RSV-attributable deaths occurred in hospitals [1]. The striking disparity between in-hospital and community deaths in low-income settings can mostly be explained by inadequate healthcare accessibility, high healthcare expenses, and restricted hospital bed capacity during an RSV epidemic. Another explanation posits that deaths might occur in children with rapidly progressive illnesses despite their initial presentation lacking signs of serious illness. The annual global expenditures for managing inpatient and outpatient cases of RSV-LRTIs in children younger than 5 years amount to approximately €5 billion, 65% of which originates from developing countries [15]. The substantial disease burden of RSV highlights the necessity for immunization programs targeting early life.

RSV typically causes seasonal outbreaks globally, with epidemics occurring from November to April or May in the Northern Hemisphere and from May to September in the Southern Hemisphere, while seasonal waves are typically associated with rainy seasons in the tropics [11, 16–18]. This variation can be attributed to the preference of RSV for cooler temperatures and higher humidity. In tropical regions, large aerosol droplets are formed due to higher humidity and stable temperatures, resulting in less variability across the year. The introduction and relaxation of NPIs during the SARS-CoV-2 pandemic and their subsequent effects on RSV circulation have demonstrated the potential of specific measures to prevent RSV infections [3–7]. NPIs have substantially affected RSV transmission by augmenting the number of RSV-naive children and diminishing population immunity against RSV [19, 20]. Growing evidence suggests the potential for medium-term negative effects through an immunity debt, in which a greater proportion of the population is susceptible to diseases after a long period of reduced exposure [4, 5, 21, 22]. This immunity debt is a particular concern for RSV, for which temporary immunity is obtained through exposure to the virus and maternal antibodies wane quickly; without seasonal exposure, immunity decreases and susceptibility to future, and potentially more severe, infections increase. In addition to NPIs, virus‒virus interactions can interfere with RSV dynamics and seasonality [23, 24]. Profound and unprecedented changes in RSV seasonality pose new challenges in tackling RSV. Ongoing monitoring of respiratory disease indicators is required to inform future healthcare system planning, and the development and use of RSV immunoprophylactic interventions should be considered.

### Clinical features

*Recommendations*: clinicians should pay close attention to infants and young children with RSV infections, especially those at high risk, who are often severely affected by LRTIs that manifest as bronchiolitis and peak 2–4 days after onset. RSV can lead to extrapulmonary manifestations, such as central nervous system infections.

The clinical manifestations of RSV infections in children widely vary in severity according to age. Infants and young children are usually severely affected by potentially life-threatening LRTIs manifesting as bronchiolitis and/or pneumonia, whereas older children typically exhibit mild upper respiratory tract infections [25–27]. When diagnosing bronchiolitis, it should be taken into account that symptoms usually peak 2 to 4 days after onset, during which time symptoms of upper respiratory infections (e.g., fever, nasal congestion, runny nose) subside but manifestations such as shortness of breath, nasal swelling, intercostal or supraclavicular contractures, use of accessory respiratory muscles, and grunts are incredibly exacerbated [28]. A hallmark characteristic is a minute-to-minute variation in clinical findings [29]. On auscultation, crackles with recurrent wheezing may be the predominant feature of bronchiolitis. Most children with bronchiolitis have either normal radiographs or radiographic findings consistent with simple bronchiolitis, such as peribronchial thickening, hyperinflation, and atelectasis [29]. The severity of clinical manifestations also varies considerably depending on whether the infection is primary or secondary. Almost all children have been infected with RSV by the age of 2 years and repeated infections are common throughout life. LRTIs usually occur with initial infections and may be present in more than 50% of secondary infections [30–33]. Although the severity of the disease decreases after the third infection, approximately a quarter of patients exhibit symptoms of LRTIs [33]. Infants aged 2–6 months are at the highest risk of developing RSV-LRTIs [30–32]. RSV infections cause inflammation that leads to airway obstruction and bronchial smooth muscle spasms. Apnea occurs in up to 20% of infants and young children, predominantly preterm infants, and may be the predominant symptom in infants admitted to the hospital. The relative immaturity of ventilation control may contribute to its pathogenesis [26, 34, 35]. Children with severe RSV infections may develop respiratory failure, necessitating admission to intensive care units (ICUs) or the need for ventilatory support [36]. The risk factors associated with severe disease include preterm birth (delivery at < 12 weeks of gestation), chronic lung disease of prematurity, and hemodynamically significant congenital heart disease [37]. A multicenter retrospective study examining risk factors associated with severe RSV infections showed that 53% of children admitted to the pediatric ICU (PICU) were classified as having a high risk for severe RSV infections [37]. This study revealed that hemodynamically significant congenital heart disease emerged as the predominant risk factor, with chronic lung disease, neuromuscular disease, congenital airway defects, and preterm birth following suit in terms of prevalence. RSV infections can affect other organs beyond the respiratory system. The central nervous system may also be involved, leading to diseases including central apnea, epilepsy, RSV encephalopathy, RSV encephalitis, and RSV meningitis. A systematic review and aggregated case series of 155 individual cases from 26 countries in 2021 revealed that a range of 1.2%–6.5% of children with RSV infections exhibit symptoms of acute encephalitis or encephalopathy [38]. Seizures were the most frequently reported neurological feature in this study (127/150, 85%), and RSV was detected in the central nervous system in 12 cases [38]. Moreover, RSV infections have the potential to cause myocardial injury, arrhythmias, myocarditis, and possibly fulminant myocarditis [39–41]. Additional extrapulmonary manifestations, such as rash, hyponatremia resulting from increased secretion of antidiuretic hormone, and hepatitis, have also been reported in children with RSV infections [40].

### Laboratory diagnosis

*Recommendations*: polymerase chain reaction-based assays have emerged as the mainstream diagnostic technique for RSV infections in children owing to their excellent sensitivity, specificity, and rapid turnaround time.

There are four main ways to diagnose RSV (Table 1): molecular detection using nucleic acid amplification techniques, rapid antigen detection tests (RADTs), direct immunofluorescence assays (DFAs), and virus culture. Viral culture has previously been considered the gold standard for RSV diagnosis given its excellent specificity. Nonetheless, its limited sensitivity, labor-intensive requirements, and long assay duration impose constraints on the practical application of virus culture. While virus culture as a diagnostic test has largely been superseded by molecular and antigenic testing, cultivation is still required to obtain viruses for phenotypic analysis and as controls for other assay types. Serological assays are mostly employed in seroepidemiological studies and research, but their utility in diagnosing acute RSV infections in clinical settings is limited [42]. Children’s endogenous serological responses are less detectable or distinguishable in the presence of maternally derived or preexisting antibodies [43–46]. RSV antigen detection by RADTs via antigen capture and by DFAs via monoclonal antibodies for antigen detection in infected cells are both less sensitive than quantitative reverse transcription polymerase chain reactions (qRT‒PCRs) [47–54]. They are prone to higher false-positive results owing to cross-reactivity with similar proteins of related viruses, such as human metapneumovirus, and higher false-negative results, mainly owing to antigenic variation among viruses [55]. RADTs are still employed because they are less costly and require less time, expertise, and maintenance than qRT‒PCRs. Nevertheless, the key advantage of RADTs, namely, their faster turnaround time, has been challenged by molecular point-of-care tests (mPOCTs), which are gaining popularity in clinical laboratories and offer a turnaround time comparable to that of RADTs but with the performance of qRT-PCRs [56]. Currently, qRT-PCR-based assays have emerged as the mainstream diagnostic techniques for RSV infections in children owing to their remarkable sensitivity (86.4%–100%) and specificity (97.7%–100%) and are widely used instead of virus culture [48, 56–60]. Certain nucleic acid amplification assays allow for discrimination between RSV-A and RSV-B. However, they are more expensive and not always available compared to antigen detection. Elevated temperatures, freeze‒thaw cycles, and changes in pH adversely affect viral infectivity [61]. Specimens should be maintained at 4 °C for testing within 1–2 days, and those that cannot be tested within this timeframe should be stored at − 70 °C or below for subsequent testing [57].

**Table 1:** Detection methods for RSV infections

Methods	Test type	Sensitivity	Specificity	Test time	Notes
Nucleic acid amplification techniques	Rapid molecular tests	90.6%–100%	99.4%–100%	13 min–1 h	The mainstream diagnostic techniques; concerns about oversensitivity in detecting clinically insignificant low-level viral titers; require evaluation of assay performance by external quality assessment
	Molecular tests	86.4%–100%	97.7%–100%	1–8 h	
Antigen detection	RADTs	72.4%–90%	89.5%–100%	< 0.5 h	Limited sensitivity in older models; automated tests offer better performance; negative specimens should be verified with another method
	DFAs	93.5%–94.1%	96.8%–99.8%	1–4 h	Requires a swab that allows for an appropriate number of epithelial cells to be collected
Virus culture	Shell vial culture	–	–	1–2 d	Traditionally considered the gold standard; many factors affect the success of virus isolation
	Virus culture	–	–	3–7 d	

*RSV* respiratory syncytial virus, *RADTs* rapid antigen detection tests, *DFAs* direct immunofluorescence assays

*Recommendations*: appropriate collection timing and specimen quality greatly influence the sensitivity of virus detection. We advise nasopharyngeal swab specimen collection preferably in the first 4 days following disease onset, if conditions permit, for molecular or antigenic detection of RSV infections.

The timing of sampling directly affects the accuracy of a laboratory diagnosis. For the highest sensitivity, we advise collecting specimens preferably in the first 4 days following disease onset. The duration of RSV shedding in outpatients averages 9.8 ± 4.8 days in adults and can be even longer in children (up to 30 days), particularly in very young age and immunocompromised patients (median, 16 days) [62–64]. The number of positive samples drops more rapidly with time after disease onset when using antigen detection compared with qRT‒PCR, indicating that the sensitivity of antigen detection is primarily high only within the initial days after disease onset [65]. Notably, diagnostic sensitivity fell by varying degrees when nucleic acid or antigen testing was available earlier [66]. Hence, it is imperative to take into account factors such as the time of sampling from disease onset, age, immunological status, and the specific technologies employed for detection when interpreting diagnostic results.

Airway epithelial cells are the primary targets of RSV infection in vivo. The anatomical site of specimen collection is an important factor influencing the sensitivity of diagnostic laboratory testing. Samples from nasopharyngeal swabs are more sensitive than those from oropharyngeal swabs because of the higher viral load in the nasopharynx than in the oropharynx [67, 68]. Furthermore, nasopharyngeal specimens are more sensitive to RSV than mid-turbinate specimens [69–71].

### Treatment

*Recommendations*: supportive care to improve airway patency, ensure oxygen demand, and guarantee adequate feeding and hydration is the mainstay of treatment for children with RSV infections.

Airway obstruction and atelectasis in bronchiolitis can result in hypoxemia, which can be relieved by oxygen supplementation. Currently, there is a paucity of evidence supporting the pulse oxygen saturation (SpO_2_) cutoff value for initiating oxygen supplementation. The American Academy of Pediatrics (AAP) practice guideline suggests SpO_2_ 90% as the threshold for initiating oxygen supplementation [72]. The British National Institute of Health and Care Excellence advises the same SpO_2_ threshold for initiating oxygen supplementation for children aged > 6 weeks as the AAP, whereas a 92% SpO_2_ threshold is advised for infants aged < 6 weeks or children of any age with underlying health conditions [73]. In China, it is advised to initiate oxygen supplementation when SpO_2_ remains continuously below 90%–92% [73, 74]. A randomized controlled trial (RCT) found that using an oxygen saturation threshold of 90% (compared with a threshold of 94%) for determining oxygen administration and hospital discharge significantly reduced the need for supplemental oxygen, length of stay, and readmission rates [75].

Respiratory support for infants and young children with bronchiolitis is generally provided in a stepwise fashion. Traditionally, hypoxemia has been treated by administering low-flow or standard subnasal oxygen via nasal prongs at maximum ceiling rates of 2–3 L/minute or via face masks at maximum ceiling rates of 15 L/minute [76]. Infants who are at risk of progressing to respiratory failure typically undergo advanced management with humidified high-flow nasal cannula oxygen (HFNC) and/or nasal continuous positive airway pressure ventilation (nCPAP) before resorting to tracheal intubation. HFNC enables the administration of high flows (up to 2–3 L/kg/minute with a maximum of 60 L/minute) with humidification to improve patient tolerance [77]. Evidence for the efficacy of HFNC therapy is predominantly observational, with studies documenting improved respiratory parameters and reduced intubation rates following the adoption of HFNC therapy [78]. One multicenter randomized trial suggested that nCPAP may be more effective than HFNC as the initial respiratory support for young infants admitted to a PICU for moderate-to-severe acute viral bronchiolitis (relative risk, 1.63) [79]. Nonetheless, there were no significant differences between HFNC and nCPAP for time to liberation from respiratory support (52.9 h for HFNC vs. 47.9 h for nCPAP) [80]. As the HFNC system is easily set up and well tolerated by patients, it has been widely adopted in the PICU and for the interhospital transport of critically ill children and is considered an effective means of providing postextubation support, particularly in underserved settings [81]. However, in children with hemodynamic instability, intractable apnea, or loss of protective airway reflexes, clinicians should prioritize initial endotracheal intubation over the use of HFNC or nCPAP [82].

Superficial nasal aspiration to improve airway patency, oxygen saturation, and feeding is appealing given that infants are obligatory nasal breathers. Nevertheless, there is a lack of RCTs that have investigated the effects of nasal aspiration on bronchiolitis. The available evidence of limited quality indicates a potential association between deep nasal aspiration and adverse outcomes as well as an extended duration of hospitalization [83]. Further evaluation of the benefits of nasal aspiration is needed.

Infants hospitalized with RSV bronchiolitis often experience difficulty maintaining adequate hydration to ensure the stability of internal water and electrolyte levels owing to nasal congestion or hypoxemia related to lower airway disease. Therefore, maintaining proper hydration remains a fundamental aspect of medical treatment. For children who can tolerate enteral feeding, strategies to maintain hydration include frequent feedings in smaller portions or orogastric or nasogastric feedings [84–86]. A multicenter randomized trial in Australia and New Zealand comparing nasogastric hydration with intravenous hydration in infants aged 2–12 months revealed no significant differences in terms of length of stay, adverse events, ICU admission, or the need for ventilation but a higher successful first-attempt insertion rate in the former [86]. Plasma antidiuretic hormone levels may be elevated in certain instances, resulting in fluid retention and hyponatremia [87]. If intravenous fluids are administered, isotonic fluids are preferred to prevent hyponatremia [73].

*Recommendations*: the role of nebulized 3% hypertonic saline in children with RSV-LRTIs is controversial. However, according to the latest meta-analysis, it improves clinical symptoms, reduces hospitalization rates, and shortens the length of stay.

Nebulized hypertonic saline (HS) solution at a concentration of 3% or more has been found to hydrate the airway surface, reduce airway edema, improve mucus clearance, and exhibit good tolerability with few adverse effects [88]. Numerous rigorous studies have been undertaken to investigate the efficacy of treatment with HS in children with RSV-LRTIs, but they have yielded conflicting findings in certain instances. Multiple RCTs have demonstrated no differences in admission rate and average length of stay between the nebulized 3% HS and control groups [89–92]. In contrast, the latest systematic meta-analyses from RCTs indicated that HS nebulization improved clinical symptoms, reduced hospitalization rates, and shortened the length of stay [93, 94]. A systematic analysis enrolling 4186 children from 150 RCTs and 32 publications showed that 3% HS nebulization was effective in reducing the length of stay and symptom severity in children with acute bronchiolitis [94]. A meta-analysis pooled 35 RCTs and found that HS nebulization significantly reduced length of stay and hospitalization rates, as well as improved 24-, 48-, and 72-hour clinical severity scores in children with bronchiolitis [93]. Moreover, there were no significant differences between the effects of HS at a concentration of 3% and those at concentrations exceeding 3%. Therefore, it can be considered a treatment option for children with RSV-LTRIs.

*Recommendations*: antiviral medications are not typically advised for previously healthy children with RSV-LRTIs, considering their safety and effectiveness. Nonetheless, the administration of antiviral drugs, such as ribavirin, may yield favorable outcomes in children with immunodeficiency. New, promising antiviral candidates are under clinical trials.

In light of the significant global burden of RSV infections, considerable resources have been dedicated to the development of antiviral medications aimed at directly impeding viral replication. Nonetheless, the number of antiviral medicines approved for clinical usage is limited due to either adverse effects or the development of resistance [95]. Ribavirin, a well-established antiviral agent with broad efficacy against RNA viruses, is not frequently employed in treatment because of concerns over its potential carcinogenic and teratogenic effects as well as detrimental outcomes in fetal development. However, it is worth noting that these deleterious effects have only been observed in rodent models instead of in primates or human beings [96]. Available data regarding the safety of ribavirin in pediatric patients are limited [97]. There is a limited amount of research with suboptimal quality and small sample sizes that has examined the impact of ribavirin on RSV infections in children. In a systematic review conducted in 2022, the available data from 10 observational studies encompassing both adult and pediatric populations, as well as an RCT involving healthy infants, were synthesized. The findings of this review indicated that the administration of ribavirin did not yield significant reductions in mortality rates, proportions of mechanically ventilated patients, viral load levels, or rates of bacterial coinfections among previously healthy individuals with RSV infections. The available evidence exhibits substantial heterogeneity, covering variations in the routes of administration, doses and durations of ribavirin therapy. Hence, it is not advisable to administer ribavirin to pediatric patients without underlying health conditions. Nonetheless, ribavirin may serve as an alternative treatment for RSV infections in immunocompromised patients. A study found that in patients with hematological malignancies and hematological stem cell transplants, mortality was significantly reduced when ribavirin was administered, with a relative risk of 0.32 [97].

Several novel antiviral drugs are under investigation. Ziresovir (AK-0529), a potent, selective, and orally bioavailable RSV F protein inhibitor that primarily blocks the entry of the virus into the host cell, is currently the only direct-acting antiviral agent against RSV that has completed a phase 3 registration clinical study. The clinical study met the primary and key secondary endpoints, showing significant clinical improvement in RSV bronchiolitis accompanied by a marked reduction in viral load and a favorable safety profile. Along with other novel antivirals, such as RV521, JNJ-53718678, and EDP-938, they showed good pharmacokinetics and potent antiviral effects in phase 2 and 3 clinical trials [98–101]. The nebulized RSV antiviral drug ALX-0171 reduced the RSV load in mid-nasal turbinate samples but did not provide significant relief from clinical symptoms [102].

*Recommendations*: administration of nebulized or systemic glucocorticoids is not advised as a routine treatment for children with RSV-LRTIs due to the absence of significant benefits in both short- and long-term outcomes.

Considerable studies have been undertaken to investigate the efficacy of glucocorticoids in the treatment of children with RSV infections. These studies have yielded findings pertaining to various outcomes, including the remission of clinical symptoms, hospitalization rates, length of stay, and long-term prognosis. Given that RSV is the predominant pathogen in the pathogenesis of bronchiolitis, a large portion of the studies on RSV have relied on investigations conducted on individuals diagnosed with bronchiolitis of unidentified etiology. However, the administration of glucocorticoids by different routes, doses, and formulations does not yield the expected outcomes [103–108].

High-quality evidence from RCTs consistently suggests that both nebulized and systemic glucocorticoids with different dosages, durations, and types do not prevent hospital admission and do not improve short- and long-term outcomes in children with RSV-LRTIs. Therefore, it is generally not advisable to administer glucocorticoids, notwithstanding their potential efficacy in particular populations. As mentioned in the section on bronchodilators, oral dexamethasone combined with salbutamol nebulization has been shown to reduce the length of stay in a select subset of children with bronchiolitis with eczema or a family history of asthma in a first-degree relative [109].

*Recommendations*: administration of bronchodilators, such as the beta-2 agonist salbutamol, is not advised as a routine treatment for children with RSV-LRTIs.

There is no observed benefit in administering inhaled bronchodilators, such as beta-2 agonists alone or in combination with other therapies, to children with wheezing after RSV infections [110, 111]. A 2014 Cochrane systematic review assessed the effects of bronchodilators on infantile bronchiolitis and concluded that the administration of salbutamol did not significantly reduce hospital admissions or shorten the length of stay [111]. Another 2020 systematic review and meta-analysis of 13 RCTs with 977 participants showed that treatment of infantile bronchiolitis with salbutamol resulted in increased respiratory and heart rates but did not improve clinical severity scores, length of stay, or oxygen saturation in infants with bronchiolitis [110]. Hence, it is not advisable to propose the administration of salbutamol in the treatment of pediatric patients with RSV-LRTIs due to its lack of efficacy in improving clinical outcomes and its potential for adverse effects. A recently published retrospective study analyzed children diagnosed with acute bronchiolitis in December during four epidemic periods, enrolling 1767 children [112]. The study showed that with the decreasing rate of salbutamol administration over time, hospitalization rates could be reduced without changing readmission rates within 72 hours, further supporting the unnecessary administration of salbutamol. The administration of magnesium sulfate as a bronchodilator is also not associated with significant improvements in the bronchiolitis severity score or length of stay [113, 114].

Based on a rigorous evidence-based medical rationale, international guidelines rarely advise the routine administration of bronchodilators in managing bronchiolitis [72]. Infected children exhibit a high degree of heterogeneity in their clinical presentation, immune response, and molecular immune profile and show different responses to treatment options, which raise the requirement for phenotype-specific treatment strategies. An RCT enrolled 200 children with bronchiolitis and showed that for children with eczema or a family history of asthma in a first-degree relative, the administration of oral dexamethasone combined with salbutamol nebulization reduced the time to discharge from 27.1 hours to 18.6 hours. Patients with clinical features, such as eczema or a family history of asthma in a first-degree relative, may benefit from salbutamol combination therapy [109]. A study conducted at Sapienza University, Rome, Italy, prospectively enrolled 312 healthy full-term infants hospitalized for bronchiolitis during 12 epidemic seasons, with diagnosis confirmed by positive RSV nucleic acid in nasopharyngeal washings and sequencing of RSV genotypes [115]. Stratification data based on genotypes revealed that low-virulence RSV genotypes preferentially caused bronchiolitis in infants who might have a genetic susceptibility to asthma and atopy. This specific population may be better treated with bronchodilators.

*Recommendations*: the prevalence of RSV with bacterial coinfections is low. The administration of antibiotics in children with RSV-LRTIs is generally discouraged, unless there is sufficient suspicion or definitive evidence of bacterial coinfections.

Determining the accurate prevalence of subsequent bacterial infections among infants and toddlers who are hospitalized for RSV infections poses a considerable challenge. A 9-year prospective study was conducted at the University of Rochester School of Medicine in New York involving 565 children with RSV infections, with the aim of investigating the prevalence of secondary bacterial infections in these children. The results showed that the rate of secondary bacterial infections was only 1.2% in children with RSV infections overall. Among the 352 children who did not receive antibiotics, the rate of secondary bacterial infections was found to be 0.6% [116]. However, the administration of antibiotics in the treatment of bronchiolitis continues to be substantial, estimated at about 25%, despite the well-established viral etiology of the disease and the low prevalence of subsequent bacterial infections [117, 118]. Several factors contribute to the elevated utilization of antibiotics, including the manifestation of a high fever, challenges in accurately interpreting chest radiographs, the apparent ill appearance of infants, and the concern for missing an alternative diagnosis, such as pneumonia. A systematic review conducted in 2014 by Cochrane encompassed seven RCTs that were either single-blind or double-blind to compare the effectiveness of antibiotics against a placebo or control in the treatment of bronchiolitis, involving a total of 824 children aged < 2 years with bronchiolitis, and the findings did not support the administration of antibiotics for the treatment of bronchiolitis in terms of oxygen saturation, bronchodilator application, tube feeding, wheezing, shortness of breath, feeding difficulties, fever, cough, symptom duration, readmission, and PICU admission [117]. A systematic analysis conducted in 2017 included only two RCTs and found that the administration of antibiotics did not reduce the proportion of children with persistent symptoms at follow-up, rehospitalized for respiratory disease within 6 months, or with wheezing at 6 months compared with the control group [119]. Based on the available evidence, it is not advisable to prescribe antibiotics for RSV-LRTIs. Clinicians are more concerned about scenarios necessitating the utilization of antibiotics. Limited evidence suggests that serum C-reactive protein > 60 mg/L and procalcitonin ≥ 2 µg/L can be used as diagnostic markers to identify bacterial infections in children with LRTIs and may provide guidance for the administration of antibiotics [120, 121]. Further research is necessary to establish conclusive evidence on the exact indications of bacterial coinfections and to address inquiries pertaining to the immediate and lasting advantages of antibiotics [116, 122].

### Prevention

*Recommendations*: a new strategy for preventing RSV infections: a single injection of a long-acting monoclonal antibody is advised for infants before or during the first RSV season to prevent RSV-LRTIs. Administration of intravenous nonspecific immunoglobulin is not advised as routine management for children with RSV infections.

Maternal antibodies are generally protective against neonatal RSV infections in the first weeks of life. However, these antibodies rapidly wane and vary in effectiveness [123]. The administration of monoclonal antibodies is considered a favorable approach for the prophylaxis of RSV infections due to their high pathogen specificity [124]. Palivizumab, the first licensed monoclonal antibody for RSV prophylaxis, has been granted approval by multiple countries [125]. It functions as a humanized monoclonal antibody targeting the RSV F-glycoprotein. Palivizumab is administered as five monthly intramuscular injections during the peak season to infants born before 29 weeks gestation, infants born before 32 weeks gestation with chronic lung disease of prematurity, and infants with hemodynamically significant heart disease [126]. However, the cost-effectiveness of palivizumab prevents its universal use, even among infants at high risk.

Although preterm infants and those with underlying lung or heart disease are at the highest risk for severe illness, the majority of RSV-related hospitalizations occur in healthy full-term infants [127]. Next-generation RSV prevention antibodies have been engineered with Fc mutations to extend their half-life and enable single-dose protection for all infants in an entire RSV season. The leading candidate is nirsevimab, a monoclonal antibody approved by multiple countries for the prevention of RSV-LRTIs in infants aged 0–12 months before or during their first RSV season, and in children up to 24 months of age who remain vulnerable to severe RSV disease through their second RSV season [128]. Nirsevimab demonstrated an overall efficacy of 75% in preventing the need for medical care in term and preterm infants, was effective in reducing hospitalization, and provided more prolonged protection than a placebo [129, 130]. Clesrovimab, another anti-F monoclonal antibody, is currently undergoing assessment in a phase 2b/3 trial (NCT04767373), which determines the efficacy of reducing RSV disease in healthy pre- and full-term infants [131]. Another potential long-acting antibody, trinomab, is also in the early stages of clinical trials for RSV prevention in infants [132].

The administration of intravenous immunoglobulin (IVIG) for the management of RSV infections is predominantly grounded in empirical management or case reports [133]. Limited and inconclusive evidence supports a potential beneficial effect of intravenous administration of nonspecific human immunoglobulin in patients with severe bronchiolitis and animals infected with RSV [134, 135]. However, an RCT investigating the effects of immunoglobulin therapy on RSV-LRTIs in children did not yield any significant differences between the treatment and placebo groups in terms of outcomes such as mortality, length of stay, ventilation time, oxygen dependence, or adverse events [136, 137]. There may be a small, nonenveloped, and transmissible virus in the blood donor population, although IVIG is manufactured under stringent safety guidelines. Therefore, safety concerns remain with the administration of immunoglobulin in viral infections [138]. RSV infections are primarily treated with supportive care; therefore, IVIG therapy is not advised for children with RSV infections.

*Recommendations*: nonpharmaceutical interventions remain the predominant approach for RSV prevention. High-risk populations should take nonpharmaceutical interventions to prevent RSV infections. Efficient pediatric RSV vaccines are not currently available except for monoclonal antibodies, but several vaccines are currently in clinical trials.

As RSV is spread by horizontal transmission, via saliva droplets, and through contact with contaminated objects and surfaces, NPIs (such as frequent and accurate hand hygiene, staying at home, physical distancing, and wearing masks) are the most effective and safe methods to reduce the risk of respiratory virus infections. Moreover, NPIs have a preventive effect against environmental factors that promote the spread of the virus, such as tobacco smoke, air pollution, temperature drops, and indoor crowding [139]. NPIs are cost-effective for controlling respiratory diseases. During the SARS-CoV-2 pandemic, the global deployment of NPIs has been associated with a significant decrease in the incidence of RSV compared with the past [140–142]. Wearing a mask is an important measure of NPIs and has a significant preventive effect not only on respiratory infections in healthy children but also in immunocompromised children, including those undergoing hematopoietic stem cell transplantation, as well as newborns in high-risk enclosed settings [143, 144]. Nevertheless, due to the special vulnerability of children, wearing masks also causes discomfort and side effects such as increased heart rate, headache, fatigue, attention disorders, and claustrophobia [145]. This assertion holds special validity in the case of young infants who encounter challenges when attempting to don masks. Therefore, other preventive interventions, such as staying at home or away from crowds, are advised during RSV outbreaks for young infants. During the RSV season, medical staff should strictly implement NPIs when caring for hospitalized children.

Despite the considerable disease burden of RSV infections, there are few vaccine options for preventing pediatric RSV infections [146]. Efforts to develop an RSV vaccine continue to be vigorous. Currently, there are various approaches for developing RSV vaccines, including particle-based, vector-based, live attenuated or chimeric, subunit, and mRNA vaccines [147]. However, there are no licensed pediatric RSV vaccines available for implementation in clinical settings. The development of vaccines remains uninterrupted, and failures and updates continue to occur. Encouragingly, several candidate vaccines are undergoing phase 3 trials. Maternal vaccines for infant protection are also under development [148].

### Long-term consequences

*Recommendations*: RSV-LRTIs in early childhood are associated with long-term complications, including impaired lung function, recurrent wheezing, and asthma.

Increasing evidence suggests an unequivocal association between early-life RSV-LRTIs in children and the subsequent development of asthma, recurrent wheezing, and impaired lung function [149–154]. The immune response of the body after RSV infections in infants and children, along with the influence of neuromodulatory mechanisms and the persistence of RSV leading to adaptive remodeling of airway ultrastructure, may cause airway hyperresponsiveness, which is strongly associated with the development of recurrent wheezing and asthma later in life [155–158]. A recent review of 906 patients with asthma found that viral LRTIs in infants aged ≤ 2 years were associated with an increased risk of asthma for up to 20 years thereafter (odds ratio = 5.0; 95% confidence interval, 3.3–7.5), with RSV as the predominant pathogen (83.3%) [159]. Another prospective population-based cohort study showed that the prevalence of asthma at age 5 years was higher in children with RSV infections in infancy than in children without evidence of RSV infections in infancy (21% vs. 16%) [150]. Nevertheless, it is unclear whether RSV infections are causal factors, markers of susceptibility to respiratory illness, or both [160, 161]. A systematic review and meta-analysis of 35 studies appraised the strength of evidence for a causal effect between laboratory-confirmed RSV-LRTIs before the age of 2 years and recurring wheezing illnesses [162]. The results were consistent with the hypothesis that a substantial proportion of the association between RSV infections and subsequent wheezing comes from shared genetic predisposition, with insufficient evidence to advise immunoprophylaxis for the prevention of wheezing illness. Long-term follow-up studies are needed before assuming that prevention of RSV-LRTIs can reduce recurrent wheezing or asthma.

In conclusion, RSV substantially contributes to morbidity and mortality globally in children younger than 5 years, especially during the first 6 months of life and in low- and middle-income countries. Profound and unprecedented changes in RSV epidemiology after the SARS-CoV-2 pandemic pose new challenges in tackling RSV. Healthcare professionals must address the increasing challenge of RSV in clinical practice. Here, we produced evidence-based recommendations that pertain to the diagnosis, treatment, and prevention of RSV infections. Transparency in production and reporting promotes scientific discourse and improves the usability of the consensus for clinicians. The consensus development group acknowledges that the lack of high-quality evidence for certain recommendations is a limitation of this consensus but tries to take this into consideration when formulating recommendations. Further studies orientated by clinical problems will be required to address knowledge gaps and help inform the management and prevention of RSV infections.

## Data Availability

Data sharing is not applicable to this article as no datasets were generated or analyzed during the current study.
